# Local descriptive body weight and dietary norms, food availability, and 10-year change in glycosylated haemoglobin in an Australian population-based biomedical cohort

**DOI:** 10.1186/s12889-017-4068-3

**Published:** 2017-02-02

**Authors:** Suzanne J. Carroll, Catherine Paquet, Natasha J. Howard, Neil T. Coffee, Robert J. Adams, Anne W. Taylor, Theo Niyonsenga, Mark Daniel

**Affiliations:** 1 Spatial Epidemiology and Evaluation Research Group, School of Health Sciences and Centre for Population Health Research, University of South Australia, IPC CWE-48, GPO Box 2471, Adelaide, South Australia 5001 Australia; 2 Research Centre of the Douglas Mental Health University Institute, Verdun, Québec Canada; 3 Discipline of Medicine, The University of Adelaide, Adelaide, South Australia Australia; 4 Department of Medicine, The University of Melbourne, St. Vincent’s Hospital, Melbourne, VIC Australia; 5 South Australian Health & Medical Research Institute, Adelaide, South Australia Australia

**Keywords:** Cardiometabolic risk, Food environment, Built environment, Descriptive norms, Multilevel models

## Abstract

**Background:**

Individual-level health outcomes are shaped by environmental risk conditions. Norms figure prominently in socio-behavioural theories yet spatial variations in health-related norms have rarely been investigated as environmental risk conditions. This study assessed: 1) the contributions of local descriptive norms for overweight/obesity and dietary behaviour to 10-year change in glycosylated haemoglobin (HbA_1c_), accounting for food resource availability; and 2) whether associations between local descriptive norms and HbA_1c_ were moderated by food resource availability.

**Methods:**

HbA_1c_, representing cardiometabolic risk, was measured three times over 10 years for a population-based biomedical cohort of adults in Adelaide, South Australia. Residential environmental exposures were defined using 1600 m participant-centred road-network buffers. Local descriptive norms for overweight/obesity and insufficient fruit intake (proportion of residents with BMI ≥ 25 kg/m^2^ [*n* = 1890] or fruit intake of <2 serves/day [*n* = 1945], respectively) were aggregated from responses to a separate geocoded population survey. Fast-food and healthful food resource availability (counts) were extracted from a retail database.

Separate sets of multilevel models included different predictors, one local descriptive norm and either fast-food or healthful food resource availability, with area-level education and individual-level covariates (age, sex, employment status, education, marital status, and smoking status). Interactions between local descriptive norms and food resource availability were tested.

**Results:**

HbA_1c_ concentration rose over time. Local descriptive norms for overweight/obesity and insufficient fruit intake predicted greater rates of increase in HbA_1c_. Neither fast-food nor healthful food resource availability were associated with change in HbA_1c_. Greater healthful food resource availability reduced the rate of increase in HbA_1c_ concentration attributed to the overweight/obesity norm.

**Conclusions:**

Local descriptive health-related norms, not food resource availability, predicted 10-year change in HbA_1c_. Null findings for food resource availability may reflect a sufficiency or minimum threshold level of resources such that availability poses no barrier to obtaining healthful or unhealthful foods for this region. However, the influence of local descriptive norms varied according to food resource availability in effects on HbA_1c_. Local descriptive health-related norms have received little attention thus far but are important influences on individual cardiometabolic risk. Further research is needed to explore how local descriptive norms contribute to chronic disease risk and outcomes.

## Background

Public health interventions commonly focus on modifiable individual-level risk factors such as dietary behaviour. However, individual-level risk factors are themselves shaped by environmental risk conditions, that is, properties of environmental living conditions that exacerbate a vulnerability to disease for the individuals exposed to those places [1]. Individual-level health behaviours, such as dietary choices, are one possible pathway through which local environments may influence health outcomes such as cardiometabolic risk [2]. For example, fast food intake may be influenced by the number of fast-food outlets in an individual’s residential area [3].

Environmental features can be contextual (i.e., features of areas) or compositional (i.e., aggregated characteristics of people residing within areas) [1, 4]. Both contextual and compositional features are associated with cardiometabolic risk. A comprehensive review concluded there were reasonably consistent associations reported between accessibility to a supermarket and lower body weight, and between convenience store and fast-food outlet accessibility and higher body weight [5], higher body weight being a cardiometabolic risk factor. Some studies, however, have not observed any relationship between cardiometabolic risk and features of the food environment. Others have observed counterintuitive associations. One US study among low-income women reported no associations between body mass index (BMI) or cardiovascular disease (CVD) risk and the density of grocery stores, fast-food outlets, restaurants, or minimarts [6]. Similarly, a multi-ethnic study of pregnant women in the UK observed no associations between fast-food availability (count of outlets) or accessibility (distance to nearest outlet) and BMI or obesity for non-South Asian pregnant women [7]. For South Asian pregnant women, the same study reported an unexpected negative association between fast-food availability and accessibility and BMI and obesity [7]. Explanations for null or unexpected observations need to reach beyond demographic attributions such as ethnicity and socioeconomic status (SES). It is possible that additional, broader factors, not accounted for by statistical adjustments for SES, such as norms, could shape the nature of relationships between food resources and health outcomes.

Numerous studies have investigated whether contextual features of local environments (e.g., fast-food outlets) are related to cardiometabolic risk, particularly body weight. Fewer studies have assessed the relationships between cardiometabolic risk and compositional features of local environments, beyond area-level SES. Associations between area-level SES and cardiometabolic risk are now very well established [5]. What remains to be far better investigated are the aggregated characteristics of people *beyond* area-level SES, for example, health-related norms, as they vary geographically. Local descriptive health-related norms may be important factors shaping cardiometabolic risk and disease through their effects on collective lifestyles and behaviour.

Though norms feature prominently in behavioural theories, for example the Theory of Planned Behaviour [8], norms are not always well defined within research. Social norms can be differentiated into *injunctive* and *descriptive* norms [9]. *Injunctive norms* are ‘shared rules of conduct’, that is, what *ought* to be done, while *descriptive norms* are what most people actually do [9]*.* Injunctive and descriptive norms are likely to influence individuals through different motivational processes [9, 10].

Descriptive norms can be further differentiated into *subjective* and *local* descriptive norms [11, 12]. *Subjective descriptive norms* refers to what friends and family typically do. In contrast, *local descriptive norms* are what people sharing the same spatial setting, such as a work-place or residential area, typically do. This is regardless of any emotional connection, or lack thereof, between individuals within the setting [11–13]. Local descriptive norms have been associated with littering and recycling behaviours [9, 14]. While subjective descriptive norms, such as smoking behaviour, have been explored within social networks [15], local descriptive norms have rarely been examined in relation to health outcomes.

A longitudinal study (involving 13 years of follow up) by Blok and colleagues [16], found neighbourhood prevalence of overweight/obesity predicted normal weight individuals becoming overweight/obese after accounting for individual factors and neighbourhood SES. Unfortunately, the study did not account for contextual features of the local environment, such as food availability, which may account for both prevalence of overweight/obesity and change in individual-level BMI. A recent longitudinal study using the same cohort reported on here accounted for contextual features of the physical activity environment, finding that local descriptive norms for overweight/obesity and physical inactivity predicted rising HbA_1c_ concentrations over time [17].

Local descriptive health-related norms may be important influences on clinical outcomes by predisposing individuals towards or against particular health behaviours. It is important to empirically assess the influence of such norms on individual-level health outcomes, ideally while accounting for potential confounders such as availability of health-related resources. Furthermore, while local descriptive health-related norms may act as predisposing factors for health-related behaviours, the availability of contextual resources may enable (or inhibit) such behaviour. Thus the availability of health-related resources may modify associations between local descriptive health-related norms and health outcomes that are a function of behaviour. For example, associations between a local descriptive norm for overweight/obesity and the development of cardiometabolic risk in individuals may be more pronounced in areas with greater, as opposed to lesser, fast-food availability.

Few studies have assessed contextual and compositional interaction effects in relation to important public health issues such as the rising level of cardiometabolic risk. Specifically, no study published thus far has investigated whether cardiometabolic risk is related to spatial variation in local-area norms for body weight and dietary behaviour while accounting for the built food environment, and whether any such relationship varies with food resource availability. This study assessed in a population-based biomedical cohort: 1) the influence of local descriptive norms for body weight and dietary behaviour on 10-year change in HbA_1c_ (a marker of cardiometabolic risk); and 2) whether associations between change in HbA_1c_ and local descriptive norms for body weight and dietary behaviour varied according to food resource availability.

## Methods

This study used an observational design incorporating data from a prospective biomedical cohort linked with other data sets utilising a Geographic Information System. The study was part of the Place and Metabolic Syndrome (PAMS) Project which aimed to assess the influence of social and built environmental factors on the evolution of cardiometabolic risk. The PAMS Project received ethical approval from the University of South Australia, Central Northern Adelaide Health Service, Queen Elizabeth Hospital, and South Australian Department for Health and Ageing Human Research Ethics Committees.

### Study area

**Fig. 1:**
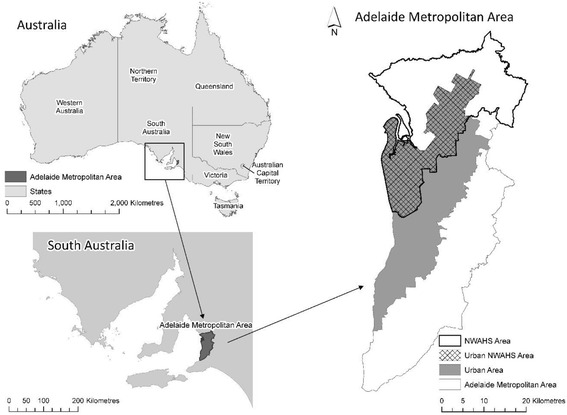
Study area – North-western region of Adelaide (urban area) (Reprinted from Social Science & Medicine, Vol. 166, Carroll, SJ, Paquet, C, Howard, N, Coffee, NT, Taylor, AW, Niyonsenga, T & Daniel, M, Local descriptive norms for overweight/obesity and physical inactivity, features of the built environment, and 10-year change in glycosylated haemoglobin in an Australian population-based biomedical cohort, pp. 233–243, 2016, with permission from Elsevier)

The baseline study area consisted of the northern and western regions of Adelaide (Fig. 1), the capital city of South Australia. These regions accounted for 38% of the city’s 1.1 million population in 2001 [18, 19] and are of particular interest due to elevated cardiometabolic risk relative to other areas [20, 21].

Associations between environments, health behaviours and outcomes may differ between urban and rural regions [22]. This study was therefore limited to urban areas only, defined as Census Collection Districts (CDs) with a population density of >200 persons per hectare [19].

### Participants

Individual-level data were sourced from the North West Adelaide Health Study (NWAHS), a 10-year biomedical cohort incorporating three waves of data collection, Wave 1 (2000–03), Wave 2 (2005–06), and Wave 3 (2008–10). The NWAHS investigated the prevalence of chronic conditions, including diabetes and cardiovascular disease, and their associated risk factors [23]. Households identified as within the study region by postcode were randomly selected from the Electronic White Pages telephone directory, and the person aged 18 years or over with the most recent birthday invited to participate in the study. Each NWAHS wave involved the collection of standardised measures using Computer-Assisted Telephone Interviews, self-report paper questionnaires, and clinic visits. Fasting blood samples were collected during the clinic visits and used to assess glycosylated haemoglobin (HbA_1c_) concentration. Written informed consent was obtained prior to each wave of data collection. Georeference points, made from participant residential addresses at each wave, enabled data linkage with other spatial datasets.

To retain cohort study participants, a multi-strategy approach was employed including consistent use of study promotional materials, newsletters and birthday cards, tracking via White Pages telephone directory and State Electoral Roll [23]. Of the 4056 Wave 1 participants, 3205 (79.0% of baseline sample) attended the Wave 2 clinic assessment and 2487 (77.6% of Wave 2 sample; 61.3% of baseline sample) attended the clinic at Wave 3. The baseline NWAHS sample was not statistically significantly different to the Adelaide metropolitan population [24] by sex, education or household income. However, older individuals (≥45 years) were over-represented in the baseline sample. Further information on recruitment and cohort profile has previously been published [23, 25].

### Measures

#### Cardiometabolic risk (outcome measure)

Glycosylated haemoglobin (HbA_1c_) concentration (%), assayed at each wave, was used to represent cardiometabolic risk. HbA_1c_ is a stable marker of glycaemic control and thus risk, reflecting 2–3 month time-averaged blood glucose levels [26]. Concentrations 6.5% or greater are indicative of diabetes [27]. However, the relationship between HbA_1c_ and cardiovascular disease (CVD) is continuous and lacking an obvious risk threshold [28].

#### Environmental measures

Environmental exposures were expressed within spatial units defined as participant-centred road-network buffers set to 1600 m (1 mile). This distance can be covered by an average adult walking at a comfortable pace of around 5 km/hour for approximately 20 min [29]. The 1600 m buffer distance has previously been used in similar studies (e.g., [30–32]) allowing for comparison of findings across studies. Smaller buffers of 1000 m were also considered but dropped due to unstable estimates of local descriptive norms associated with small counts of survey participants within buffers (see below).

Geocoded data for constructing local descriptive norms were not available prior to 2006. To temporally match data for local descriptive norms, other environmental exposures were expressed for the year 2007.

##### Local descriptive health-related norms

Local descriptive norms for overweight/obesity and insufficient fruit intake were respectively expressed as local prevalence of overweight/obesity (proportion of South Australian Monitoring and Surveillance System [SAMSS] participants per buffer classified as having a BMI ≥ 25 kg/m^2^) and insufficient fruit intake (proportion of SAMSS participants per buffer not meeting fruit intake recommendations), based on health recommendations of two or more serves per day [33, 34].

Local descriptive norms were aggregated from geocoded individual-level survey response data (adults 18 years and older), extracted from the SAMSS for the years 2006–2010. Processing of individual-level SAMSS data was performed by the data custodians to protect the confidentiality of SAMSS participants.

The SAMSS survey for which details are published elsewhere, monitors population trends in chronic diseases and risk factors [35, 36]. SAMSS participants are recruited annually across all of South Australia by simple random sampling of households from the Electronic White Pages telephone directory. The individual, of any age, with the most recent birthday is invited to participate. Overall, the response rate for SAMSS contacts during 2006–2010 was 65% with 35,830 interviews conducted across South Australia. Of the 8355 SAMSS participants interviewed during 2006–2010, 18 years and over residing within the NWAHS region, 6860 participant records were geocoded (82%); 1439 participants did not provide consent (17%) and 56 (<1%) could not be geocoded.

To maximise SAMSS participant representation within each NWAHS participant buffer, SAMSS data were pooled across survey years 2006 to 2010. To protect confidentiality and support the reliability of estimates, aggregated norms data for NWAHS buffers with fewer than 50 SAMSS participants, or less than five participants per measurement category, were not released by the data custodians. Consequently sample loss occurred which was particularly severe at the 1000 m buffer size and hence this unit was not considered further. Unstandardised prevalence rates were used following the precedent of Blok and colleagues [16]. Appropriate weightings for standardisation were unavailable at the level of the geographic buffers used, and the use of other weightings (e.g., for the Adelaide metropolitan region) may artificially reduce or inflate spatial variation.

##### Contextual features

Contextual data were extracted from the 2007 South Australian Retail Database [37]. The database catalogues shops, with information including shop location, retail activity type, and shop floor-space. Retail activity type is coded based on predominant retail activities [38]. Contextual food environment data were extracted according to these retail codes.

Food resources were classified by the authors based on these retail codes, using classifications designed by a dietician for use in a previous Australian study [39]. Fast-food outlets were defined as major fast-food franchises (e.g., McDonalds©) and independent fast-food take-away stores (e.g., fish and chips). Healthful food resources were defined as greengrocers, butchers, supermarkets (with > 200 m^2^ floor space), and health food shops. Food outlets selling a mix of healthful and unhealthful foods, with neither food group being obviously predominant (e.g., sandwich and lunch bars, bakeries, and restaurants other than those identified as fast food), were excluded from classification.

Road-network distance from NWAHS participants’ residence to food resources was calculated using Network Spatial Analyst in ArcGIS (version 9.3.1, ESRI, Redlands, California). Healthful food resources and fast-food outlets identified within 1600 m of participant residences were then summed according to type. Density measures (count/area of buffer intersected parcels in km^2^) were calculated in addition to counts.

#### Covariates

Individual- and area-level covariates were included in analytic models. Predictors of NWAHS cohort attrition were assessed using logistic regression within the analytic sample (i.e., after application of inclusion criteria as listed in Table 1). The pattern of missingness did not meet the *missing completely at random* criterion. As participants who were younger, not in the work-force, currently a smoker, and not married (or de facto) were more likely to have missing HbA_1c_ information at follow ups, these measures were included in statistical models to satisfy the analytic criterion of *missing at random* [40]. Therefore individual-level covariates included age, sex, employment status (full-time, part-time, or not in the work force), level of education (university graduate or not), marital status (married/de facto, or single), and smoking status (current smoker, ex-smoker, or never smoked). Covariates other than baseline age and sex were treated as time varying.

**Table 1:** Loss of analytic sample due to application of inclusion criteria

	Number	Reason for reduced numbers
NWAHS Wave 1	4056	-
Geocoded (Baseline)	4041	15 participants with invalid residential addresses
Residing in urban area (Baseline)	3887	154 participants outside of urban area
Did not change residential address between Wave 1 and Wave 2	3322	565 participants moved between Wave 1 and Wave 2
State Suburbs with ≥ 10 participants	3173	149 participants resided in suburbs with ≤ 9 other participants
CVD/diabetes free at baseline	2621	552 participants reported CVD/diabetes at baseline
At least one set of HbA_1c_ and individual-level covariates data	2582	39 participants did not have at least one set of complete HbA_1c_ and individual-level covariates data
Contextual features data	2213	369 participants did not have fast-food outlet or healthful food resource availability data at Wave 2
*Compositional norms data*		
Prevalence of overweight/obesity (BMI ≥ 25 kg/m^2^)	1890	323 participants lacked local descriptive overweight/obesity data
Prevalence of insufficient fruit intake (<2 serves daily)	1945	268 participants lacked local descriptive fruit intake data

Area-level education (proportion with a university degree) was selected to represent area-level SES. The use of area-level education allows interpretation of specific area-level SES relations with health outcomes (i.e., change in HbA_1c_) and comparisons with studies similarly using education to express area-level SES. Education data were extracted from the 2006 Population and Housing Census [41] at the level of CDs and further aggregated using the weighted average of values from CDs intersected by the NWAHS participant buffers. CDs, the smallest unit for which census data are available, include an average of 220 dwellings [42]. Weights were defined based on the proportion of dwellings within a CD included within the NWAHS participant buffer:

$$ BUFFE{R}_{SES}={\displaystyle \sum}\left[ C{D}_{SES} \times \frac{dwelling{ s}_a}{dwelling{ s}_b}\right] $$

where *dwellings*_*a*_ represents the number of dwellings included within a CD intersected with a buffer, and *dwellings*_*b*_ represents the total number of dwellings within a buffer. Though assuming that the distribution of the characteristic of interest (area-level education) is evenly distributed across all dwellings, this method is an improvement over assuming that the characteristic is evenly spread across the spatial unit with no recognition of the distribution of dwellings.

### Analyses

Linear multilevel models (three levels), assessed associations between environmental features and 10-year change in HbA_1c_. Level one of the model (time) regressed time-specific HbA_1c_ data on time of measurement (in years) from baseline data collection. As data collection between participant waves was unevenly spaced, with slightly different years possible within each wave, time was expressed in a continuous format from the participant’s first clinic visit. Level two (participant level) modelled associations between environmental exposures and participant baseline values of, and changes in, HbA_1c_. Included random effects allowed variation in baseline HbA_1c_ (intercept) and HbA_1c_ change (slope for time) between participants. Lastly, level three accounted for spatial clustering within State Suburbs, with a random intercept specified to allow for variations in baseline HbA_1c_ across State Suburbs. State Suburbs are formed by aggregating CDs to align with the most recent gazetted suburb at the time of the Census [19].

Four separate sets of models were constructed, with individual-level covariates included in all models. Predictor variables were added sequentially: 1) compositional norm (prevalence of overweight/obesity or insufficient fruit intake), time, and the two-way interaction between these terms; 2) context (fast food or healthful food availability), and the two-way interaction term (context x time); and 3) area-level education (covariate). Interaction terms for predictors and time (e.g., compositional norm x time) assessed the influence of the predictor (compositional norm) on *change* in HbA_1c_ over time. Additional two-way (compositional norm x context) and three-way (compositional norm x context x time) interaction terms were included in full models to test for interactions between environmental predictors in relation to baseline HbA_1c_ and *change* in HbA_1c_ respectively.

Environmental measures were standardised prior to analyses to allow comparison of their relative effects. All analyses were conducted using SAS (version 9.4, SAS Institute Inc, Cary, North Carolina). Statistical significance was set at alpha = 0.05.

## Results

Table 1 outlines sample loss due to analysis inclusion criteria. Participants who moved between waves 1 and 2 were excluded from analyses. The two analytic samples contained were 1890 and 1945 eligible NWAHS participants with local descriptive norms data for overweight/obesity and insufficient fruit intake, respectively. Participant characteristics and environmental features are summarised in Table 2. There were no notable differences between the two analysis samples. The majority (90.2%) of eligible participants were born in Australia, New Zealand or Western Europe, and the median length of follow-up was 6.84 years for both samples.

**Table 2:** Individual characteristics and environmental features for the analytic samples

Individual-level characteristic (baseline)	Overweight/obesity norm models (*n* = 1890)^a^ n (%)	Insufficient fruit intake norm models (*n* = 1945)^a^ n (%)
Length of follow-up (years)^b^	6.84 (4.59-8.36)	6.84 (4.54-8.36)
Sex (male)	840 (44.4%)	864 (44.4%)
Age in years^c^	49.9 (15.2)	50.0 (15.2)
*Ethnicity*:		
Born in Australia/New Zealand/Western Europe	1698 (90.2%)	1748 (90.2%)
*Employment*:		
Full-time employed	727 (38.8%)	747 (38.7%)
Part-time employed	348 (18.6%)	357 (18.5%)
Not in work-force	800 (42.7%)	826 (42.8%)
*Education*:		
Not university graduate	1639 (87.0%)	1689 (87.1%)
University graduate	245 (13.0%)	250 (12.9%)
*Smoking status*:		
Current smoker	325 (17.4%)	335 (17.4%)
Ex-smoker	631 (33.7%)	650 (33.8%)
Never smoked	915 (48.9%)	940 (48.8%)
*Marital status*:		
Single	663 (35.3%)	686 (35.5%)
Married/de facto	1215 (64.7%)	1247 (64.5%)
HbA_1c_ concentration (%)^c^	5.43 (0.45)	5.43 (0.45)
Environmental features	Mean (SD)	Mean (SD)
1600 m buffer area (km^2^)^b^	3.90 (3.29-4.83)	3.90 (3.29-4.84)
*Contextual features*		
Fast-food outlets (count)	5.5 (3.9)	5.5 (3.9)
Fast-food outlet density (count/buffer km^2^)	1.67 (1.26)	1.65 (1.26)
Healthful food resources (count)	4.1 (3.4)	4.0 (3.4)
Healthful food resource density (count/buffer km^2^)	1.01 (0.89)	1.01 (0.89)
*Compositional features*		
Overweight/obesity norm (BMI ≥ 25 kg/m^2^)	62.97% (2.21%)	-
n_(SAMSS participants)_ per buffer^b^	91 (72–116)	-
Insufficient fruit intake norm (<2 serves daily)	-	53.82% (6.55%)
n_(SAMSS participants)_ per buffer^b^	-	96 (76–122)
Area-level education (% with university degree)	10.19% (5.17)	10.09% (5.18)

^a^ total *n* may vary due to missing values for some variables at baseline; ^b^ median (IQR); ^c^ mean/SD

Intraclass correlations (ICC), describing the degree of similarity (or homogeneity) of the observed response within a given unit of analysis (i.e., HbA_1c_ concentration across waves for a participant) or cluster (i.e., State Suburb) were calculated from covariance parameter estimates of the three-level model with no predictors [43]. These ICCs indicated moderate correlation of HbA_1c_ at the individual level (repeated measures over time; ICC _participants_ = 0.57) and relatively low correlation at the suburb level (ICC _State Suburb_ = 0.01) consistent with previously reported levels of cardiometabolic risk clustering according to geographic area [44].

Tables 3 and 4 present the results of the four sets of multilevel models and adjusted ICCs. As environmental exposure measures (including area-level education) were standardised prior to analyses, the reported beta coefficients reflect change in HbA_1c_ concentration per one standard deviation (SD) change in the environmental exposure predictor. Means and SDs of environmental measures are provided in Table 2. Model 1 in each set included time, one local descriptive (either overweight/obesity or insufficient fruit intake) and individual-level covariates. Model fit (based on AIC and BIC) did not improve in any of the four sets of models with the inclusion of measures of food resource availability (neither fast food nor healthful food resources; Model 2). Similarly, the inclusion of area-level education at Model 3 did not improve model fit in sets of models including the overweight/obesity norm (Table 3). However, model fit did improve with inclusion of area-level education in models with the insufficient fruit intake norm (Table 4, Model 3), and area-level education was statistically significantly positively associated with baseline HbA_1c_. Lastly, the inclusion of the environmental exposures interaction term in model 4 did not improve model fit in three of the four sets of models. In the fourth set, the inclusion of an interaction term between the featured environmental predictors, namely overweight/obesity norm and healthful food resources, improved model fit.

**Table 3:** Associations between local descriptive overweight/obesity norm, food resource availability, and 10-year change in HbA_1c_

	Model 1	Model 2	Model 3	Model 4
Predictors	β (95% CI)	β (95% CI)	β (95% CI)	β (95% CI)
Overweight/obesity norm and fast-food outlet availability				
Time (in years – not standardised)	0.0332 (0.0301–0.0363)****	0.0332 (0.0301–0.0636)****	0.0332 (0.0301–0.0363)****	0.0331 (0.0300–0.0362) ****
Overweight/obesity norm	−0.0376 (−0.0575 to −0.0178)***	−0.0382 (−0.0580 - -0.0183)***	−0.0335 (−0.0571 to −0.0100)**	−0.0334 (−0.0569 to −0.0098)**
Overweight/obesity norm*time	0.0080 (0.0049–0.0110)***	0.0080 (0.0049–0.0111)****	0.0080 (0.0049–0.0111)****	0.0080 (0.0049–0.0111)****
Fast-food outlets	-	−0.0082 (−0.0281–0.0116)	−0.0096 (−0.0298–0.0106)	−0.0094 (−0.0296–0.0108)
Fast-food outlets*time	-	0.0002 (−0.0030–0.0034)	0.0002 (−0.0030–0.0034)	0.0003 (−0.0029–0.0035)
Overweight/obesity norm*fast-food outlets	-	-	-	−0.0021 (−0.0228–0.0186)
Overweight/obesity norm*fast-food outlets*time	-	-	-	−0.0021 (−0.0055–0.0014)
Area-level education (% university degree)	-	-	0.0090 (−0.0158–0.0337)	0.0089 (−0.0158–0.0336)
AIC	4286.0	4289.3	4290.8	4293.0
BIC	4327.5	4336.0	4340.1	4347.5
Adjusted ICC (participants)	0.60	0.60	0.60	0.60
Adjusted ICC (State Suburb)	0.01	0.01	0.01	0.01
Overweight/obesity norm and healthful food resource availability				
Time (in years – not standardised)	0.0332 (0.0301–0.0363)****	0.0334 (0.0303–0.0365)***	0.0334 (0.0303–0.0365)****	0.0335 (0.0304–0.0366)****
Overweight/obesity norm	−0.0376 (−0.0575 - -0.0178)***	−0.0379 (−0.0558 - -0.0180) ***	−0.0347 (−0.0582 to −0.0111)**	−0.0344 (−0.0579 to −0.0108)**
Overweight/obesity norm*time	0.0080 (0.0049–0.0110)***	0.0080 (0.0049–0.0111)****	0.0080 (0.0049–0.0111)****	0.0075 (0.0045–0.0106)****
Healthful food resources	-	0.0114 (−0.0082–0.0310)	0.0112 (−0.0084–0.0308)	0.0121 (−0.0075–0.0317)
Healthful food resources*time	-	−0.0024 (−0.0055–0.0007)	−0.0024 (−0.0055–0.0007)	−0.0027 (−0.0058–0.0005)
Overweight/obesity norm*healthful food resources	-	-	-	0.0208 (−0.0002–0.0418)
Overweight/obesity norm*healthful food resources*time	-	-	-	−0.0057 (−0.0092 to −0.0022)**
Area-level education (% university degree)	-	-	0.0062 (−0.0182–0.0306)	0.0040 (−0.0206–0.0286)
AIC	4286.0	4287.2	4288.9	4281.6
BIC	4327.5	4333.9	4338.2	4336.1
Adjusted ICC (participants)	0.60	0.60	0.60	0.60
Adjusted ICC (State Suburb)	0.01	0.01	0.01	0.01

All models adjusted for individual covariates: age (at baseline), sex, marital status, education, employment status, and smoking status; standardised (β) coefficients and 95% confidence intervals (CI); *p < 0.05; **p < 0.01; ***p < 0.001; ****p < 0.0001; *n* = 1890

**Table 4:** Associations between local descriptive insufficient fruit intake norms, food resource availability, and 10-year change in HbA_1c_

	Model 1	Model 2	Model 3	Model 4
Predictors	β (95% CI)	β (95% CI)	β (95% CI)	β (95% CI)
Insufficient fruit intake norm and fast-food outlet availability				
Time (in years – not standardised)	0.0337 (0.0307–0.0367)****	0.0337 (0.0306–0.0367)****	0.0337 (0.0307–0.0368)****	0.0335 (0.0304–0.0365)****
Insufficient fruit intake norm	0.0008 (−0.0189–0.0205)	0.0002 (−0.0197–0.0200)	0.0106 (−0.0104–0.0315)	0.0111 (−0.0097–0.0320)
Insufficient fruit intake norm*time	0.0055 (0.0025–0.0085)***	0.0055 (0.0025–0.0086)***	0.0055 (0.0025–0.0086)***	0.0055 (0.0025–0.0086)***
Fast-food outlets	-	−0.0053 (−0.0252–0.0146)	−0.0095 (−0.0293–0.0103)	−0.0096 (−0.0293–0.0101)
Fast-food outlets*time	-	0.0001 (−0.0030–0.0033)	0.0001 (−0.0030–0.0033)	0.0001 (−0.0031–0.0033)
Insufficient fruit intake norm*fast-food outlets	-	-	-	−0.0062 (−0.0271–0.0147)
Insufficient fruit intake norm*fast-food outlets*time	-	-	-	−0.0020 (−0.0054–0.0015)
Area-level education (% university degree)	-	-	0.0296 (0.0077–0.0514)**	0.0306 (0.0089–0.0524)**
AIC	4420.8	4424.5	4419.7	4421.4
BIC	4462.3	4471.2	4469.0	4475.9
Adjusted ICC _(participants)_	0.59	0.59	0.59	0.60
Adjusted ICC _(State Suburb)_	0.01	0.01	0.01	0.01
Insufficient fruit intake norm and healthful food resource availability				
Time (in years – not standardised)	0.0337 (0.0307–0.0367)****	0.0338 (0.0307–0.0368)****	0.0338 (0.0307–0.0369)****	0.0337 (0.0306–0.0367)****
Insufficient fruit intake norm	0.0008 (−0.0189–0.0205)	0.0020 (−0.0178–0.0218)	0.0123 (−0.0087–0.0334)	0.0115 (−0.0096–0.0326)
Insufficient fruit intake norm*time	0.0055 (0.0025–0.0085)***	0.0053 (0.0023–0.0084)***	0.0053 (0.0023–0.0084)***	0.0052 (0.0021–0.0082)**
Healthful food resources	-	0.0104 (−0.0093–0.0302)	0.0107 (−0.0087–0.0300)	0.0078 (−0.0121–0.0278)
Healthful food resources*time	-	−0.0015 (−0.0046–0.0016)	−0.0015 (−0.0049–0.0017)	−0.0018 (−0.0051–0.0015)
Insufficient fruit intake norm*healthful food resources	-	-	-	−0.0115 (−0.0320–0.0090)
Insufficient fruit intake norm*healthful food resources*time	-	-	-	−0.0011 (−0.0045–0.0022)
Area-level education (% university degree)	-	-	0.0280 (0.0063–0.0496)**	0.0300 (0.0083–0.0517)**
AIC	4420.8	4423.3	4419.1	4420.7
BIC	4462.3	4470.0	4468.4	4475.2
Adjusted ICC _(participants)_	0.59	0.59	0.59	0.59
Adjusted ICC _(State Suburb)_	0.01	0.01	0.01	0.01

All models adjusted for individual covariates: age (at baseline), sex, marital status, education, employment status, and smoking status; standardised (β) coefficients and 95% confidence intervals (CI); *p < 0.05; **p < 0.01; ***p < 0.001; ****p < 0.0001; *n* = 1945

In Models 1–3, lesser overweight/obesity norm was statistically significantly associated with greater baseline HbA_1c_ concentration (β = −0.03 to −0.04 depending on model; i.e., a 2.21% [1SD] increment in overweight/obesity prevalence was associated with a −0.03% to −0.04% lower HbA_1c_ concentration). Insufficient fruit intake norm, fast-food outlets, and healthful food resources were not associated with baseline HbA_1c_. HbA_1c_ increased over the 10-year follow-up period (time was statistically significantly positively associated with HbA_1c_ concentration in all models) with an increase in HbA_1c_ concentration of 0.03% per year. Statistically significant positive time x norm interactions indicate that greater prevalences for the overweight/obesity norm, and greater insufficient fruit intake norm, were each associated with greater rates of rising HbA_1c_ over time (e.g., in model 3 with fast food availability: overweight/obesity norm x time β = 0.008 indicating that a 2.21% [1SD] increment in overweight/obesity prevalence was associated with a further 0.008% increase in HbA_1c_ per year).

Fast-food outlets and healthful food resources were not associated with change in HbA_1c_ over time. There were no statistically significant two-way (local descriptive norm and food resource availability) interactions related to baseline HbA_1c_ concentration.

**Fig. 2:**
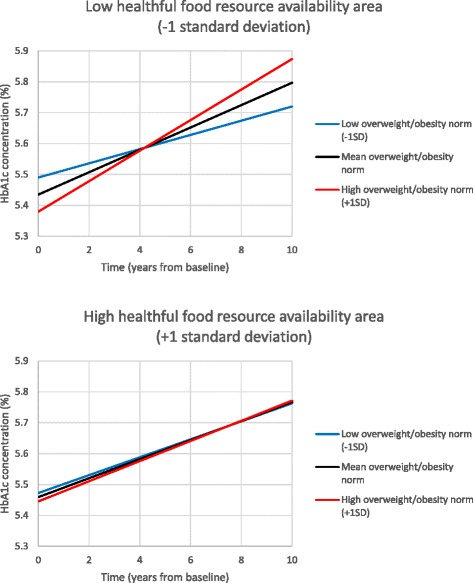
Associations between local descriptive overweight/obesity norms and HbA_1c_ trajectories according to healthful food resource availability

The three-way interaction of the overweight/obesity norm x healthful food resource x time was statistically significantly associated with HbA_1c_ (β = −0.0057 [95% CI −0.0092 to −0.0022], *p* = 0.001). The effect of healthful food resource availability on the relationship between local descriptive overweight/obesity norm and the trajectory of HbA_1c_ is shown graphically in Fig. 2. The figure shows that greater healthful food resource availability reduced the impact of the overweight/obesity norm on increasing HbA_1c_ concentration. Models including the food environment measures as density rather than count measures found similar results (not reported here).

## Discussion

Few studies have examined the influence of local descriptive health-related norms on trajectories of individual health outcomes. This study found that local descriptive norms, operationalised as the prevalence of local residents being overweight/obese or not meeting fruit intake recommendations, were each associated with the rate of increase in HbA_1c_ levels over 10 years. These relationships were robust to the inclusion of contextual measures (fast-food outlet and healthful food resource availability), area-level education, and individual-level demographic and smoking information. Fast-food outlet and healthful food resource availability were not statistically significantly associated with change in HbA_1c_ in this sample and region. However, greater healthful food resources reduced the unhealthful influence of the overweight/obesity norm on the rate of increase in HbA_1c_. This observation supports the premise that the availability of food resources can modify relationships between local descriptive health-related norms and health outcomes.

Associations between *subjective* descriptive norms and individual health-related outcomes have previously been reported for social networks. A longitudinal (32 years) social network study found associations between norms for overweight and an individual becoming overweight [45]. Similarly, the dietary norms of peers are related to individuals’ diet and dietary intentions [46, 47]. The influence of geographically defined (i.e. *local*) descriptive norms on individual cardiometabolic risk has rarely been evaluated. One study found that the odds of a Dutch adult becoming overweight/obese over 13-years of follow-up increased with greater prevalence of neighbourhood overweight/obesity in models adjusted for age, sex, education and neighbourhood deprivation [16]. Similarly, an analysis using the same cohort as reported on here, documented associations between greater local descriptive overweight/obesity and physical inactivity norms, and increasing HbA_1c_ over time in models adjusted for walkability, availability of public open space, area-level education and individual-level covariates [17]. No study has thus far reported the influence of local descriptive dietary norms on individual health outcomes. The current study’s findings, along with those of the two referenced studies, support the notion that local descriptive norms influence individual-level health outcomes. However, more research in different regions and populations is needed to replicate these results.

Behavioural theory suggests we imitate the behaviours of others, whether informed by direct viewing or by other informational sources [48, 49]. It may be that by observing locality-based body weight (i.e., the local descriptive body weight norm) an individual determines what they consider to be a socially acceptable body weight, and that the norm overrides any known health consequences associated with a larger body size. Therefore, exposure to greater prevalence of overweight or obese persons may reduce motivation to follow health recommendations relating to diet and body weight. Interestingly, the associations between insufficient fruit intake norm and change in HbA_1c_ were similar to those for the overweight/obesity norm. The fruit intake of other residents is unlikely to be easily observed, unlike local body weight norms. As such, it is difficult to understand how the eating behaviour of nearby residents may influence individuals. Norms for overweight/obesity and insufficient fruit intake were moderately correlated (rho = 0.37, *p* < 0.0001) which may partly explain these findings. It is also possible that the similarities in results reflect broader influences such as the formation of geographically defined collective lifestyles, the expression of a shared way of relating and acting in a given environment [50, 51].

Intervention strategies previously applied to reduce smoking behaviour could be adapted for use by initiatives to improve dietary behaviour. Smoking intervention has successfully changed attitudes to smoking, pushing the norm towards non-smoking due to policy intervention strategies such as increased pricing, reduced availability and limitations as to where one can smoke [52]. Similar manipulation of the food environment may assist in changing norms relating to diet behaviour and weight, particularly where norms are most unhealthful. Moreover, psychology research has shown that information on the eating behaviours of others can influence both the food selection and quantity of food consumed [53]. As such, descriptive norms information could be used to encourage increased fruit and vegetable intake [54].

This study found no association between fast-food outlet or healthful food resource availability and change in HbA_1c_. Findings from previous studies indicate mixed results in this regard [55]. Some studies report greater fast-food outlet availability as associated with: greater weight status [56, 57]; an increase in systolic and diastolic blood pressure over 1 year in low walkability neighbourhoods [58]; and mortality and hospital admissions for acute coronary syndromes [59] in models adjusted for individual and area-level covariates. Other studies of fast-food outlet availability have reported no significant association with weight status [31, 60, 61]. Still other research suggests that relationships between fast-food availability and fast-food consumption [62] and cardiometabolic risk [63] are complex, being moderated by individual psychological dispositions. Similarly, when regarding healthful food resources, some studies have found associations between healthful food availability and lower 5 year diabetes incidence [64], and greater supermarket availability and reduced odds of obesity [56]. Other studies, like ours, have not observed any association between the availability of healthful food resources and cardiometabolic risk, or have observed associations in an unexpected direction (e.g., [55, 61]).

Food resource availability is largely viewed to function as an enabler (or conversely, a barrier, where unavailable) to obtaining and consuming desired foods. Whilst different local areas within our study region are likely to have different availabilities of food resources, all might nevertheless provide access sufficient as not to unduly limit individual dietary choices or a capacity to obtain desired foods. The median number of fast-food outlets in a buffer was five (IQR 3–8), suggesting that fast food was readily available across the region and lack of access would not generally be a barrier to obtaining fast food, if desired.

Though food resource availability was not associated with change in HbA_1c_ over time, healthful food resource availability modified the association between the overweight/obesity norm and change in HbA_1c_ over time. In areas with a greater availability of healthful food resources, the impact of a greater overweight/obesity norm on rising HbA_1c_ was reduced. Conversely, where there was a lesser availability of healthful food resources, the rate of increase in HbA_1c_ due to a greater overweight/obesity norm was amplified. Interactions between environmental features in relation to cardiometabolic risk have rarely been studied [5]. No previous studies have reported on the presence or absence of interactions between local descriptive health-related norms and the contextual food environment in relation to HbA_1c_ or other health outcomes. Further study of the interactive, joint effects of contextual and compositional risk conditions on chronic disease outcomes is required to inform strategies for intervention design and targeting. Local environments that predispose *and* enable individual health behaviours will be more supportive of health than environments that support only one or the other set of factors. Attention to local health-related norms (to predispose healthful behaviour) together with the provision of sufficient resources (to enable healthful behaviour) is needed to reduce chronic disease outcomes [65, 66].

It is necessary also to develop an understanding of how local descriptive norms are shaped. Intervention strategies intending to change local norms will need to be assessed for effectiveness. Appropriate intervention strategies may need to differ according to the level of the current descriptive norm. Applying Rogers’ Diffusion of Innovations theory [67], where the local prevalence of a positive behavioural norm is low, intervention strategies might prioritise the targeting of early adopters. Conversely, where the local prevalence of a positive behavioural norm is moderate, targeting laggards may be more appropriate. Strategies might further differ depending on the target group. For example, strategies aimed at early adopters could involve health education campaigns that appeal to values, attitudes and beliefs which predispose behaviour, while those targeting the early majority could focus on enabling mechanisms such as the provision of healthful food at affordable cost. Further research is necessary to empirically measure and to implement interventions to shape and apply healthful norms.

### Strengths and limitations

Strengths of this study include the use of a 10-year population-based cohort with three waves of data including clinical measures. The longitudinal design supports causal inference through temporality of measures [2]. However, the positive longitudinal associations between the local descriptive overweight/obesity norm and increasing HbA_1c_ differed from the cross-sectional results which indicated an inverse association between the overweight/obesity norm and baseline HbA_1c_. Sub-analyses indicated that these unexpected cross-sectional findings were carried primarily by older participants and that age adjustment did not fully remove the impact of these influences. Emphasis should be on the longitudinal findings as the cross-sectional results are likely spurious.

The outcome (HbA_1c_) was clinically measured, avoiding self-report bias. However, individual demographic and smoking information were self-reported, and the local descriptive norms were aggregated from self-report survey data with the consequent possibility of self-report bias.

The methods and data sources used to operationalise the environmental exposures were strengths of this study. The contextual food environment was represented using objective measures extracted from a database constructed from data collected by field surveyors [37]. Local descriptive norms data were derived from a separate survey, thus avoiding same-sample bias [68]. Environmental exposures were defined using ego-centred road-network buffers, as has been previously recommended [69], with local-area education expressed using a spatial unit designed to closely match with these road-network buffers. The use of differently sized road-network buffers would have added to this research, however this was not possible, as previously outlined. It is also important to note the possibility that self-selection into neighbourhoods may have influenced this study’s results. This, however, is of greater concern in cross-sectional studies than those with a longitudinal design [70].

Lastly, a basic premise of this study is that people are influenced by their *local* residential environments and their opportunities to access resources within these areas. This does not account for time spent proximal to their place of residence, and opportunities and exposures provided within the work-place and other destinations, or while commuting. The influence of local residential exposures may vary according to time spent in the local residential area, which may itself relate to individual-level sociodemographic factors and lifestyle choices. Older individuals, or those caring for young children at home, may spend more time close to home and thus be more strongly influenced by local environment exposures. Future research will use technologies such as GPS tracking to assess time spent within different geographies for individuals. Consideration of transport modes may also be important. Car ownership may modify relationships between residential exposures and health outcomes. In the current study region, cars are the predominant mode of transport though public transport options are available and streets are generally walkable with adequate footpaths provided. These may be important factors to consider in future studies.

## Conclusion

Local descriptive body weight and dietary norms reflect compositional population characteristics. Food resource availability reflects context. The assessment of compositional norms in relation to health outcomes has rarely been investigated. This longitudinal study found only compositional norms, not food resource availability, to be associated with 10-year change in HbA_1c_. However, the availability of healthful food resources modified the relationship between the local descriptive overweight/obesity norm and rate of change in HbA_1c_.

Research in different populations and regions is recommended to replicate these results. It is also recommended that future research investigate how compositional norms may be shaped, and the mechanisms through which compositional norms influence individual health outcomes. The findings of this study suggest that compositional norms should be considered in intervention strategies targeting cardiometabolic risk.

## Abbreviations

AIC: Akaike information criterion
BIC: Bayesian information criterion
BMI: Body mass index
CD: Census Collection District
CI: Confidence interval
CVD: Cardiovascular disease
HbA_1c_: Glycosylated haemoglobin
ICC: Intraclass correlations
IQR: Interquartile range
NWAHS: North West Adelaide Health Study
PAMS Project: Place and Metabolic Syndrome Project
SAMSS: South Australian Monitoring and Surveillance System
SD: Standard deviation
SES: Socioeconomic status
